# Acupuncture therapies for cancer-related fatigue: A Bayesian network meta-analysis and systematic review

**DOI:** 10.3389/fonc.2023.1071326

**Published:** 2023-03-27

**Authors:** Hao Tian, Yunhui Chen, Mingsheng Sun, Liuyang Huang, Guixing Xu, Chunyang Yang, Qin Luo, Ling Zhao, Zheng Wei, Fanrong Liang

**Affiliations:** ^1^ College of Acupuncture and Tuina, The 3rd Teaching Hospital, Chengdu University of Traditional Chinese Medicine, Chengdu, Sichuan, China; ^2^ College of Basic Medicine, Chengdu University of Traditional Chinese Medicine, Chengdu, Sichuan, China; ^3^ College of International Education, Chengdu University of Traditional Chinese Medicine, Chengdu, Sichuan, China; ^4^ Clinical Research Center for Acupuncture and Moxibustion in Sichuan Province, Chengdu, Sichuan, China; ^5^ Traditional Chinese Medicine Department, People’s Hospital of Deyang City, Deyang, Sichuan, China

**Keywords:** acupuncture, acupuncture therapy, cancer-related fatigue, network meta-analysis, systematic review

## Abstract

**Background:**

Cancer-related fatigue (CRF) is one of the most commonly reported symptoms impacting cancer survivors. This study evaluated and compared the effectiveness and safety of acupuncture treatments for CRF.

**Methods:**

We searched PubMed, Embase, Web of Science, Cochrane Library, China Biology Medicine China National Knowledge Infrastructure, China Science and Technology Journal Database, and WanFang Database from inception to November 2022 to identify eligible randomized controlled trials (RCTs) comparing acupuncture treatments with sham interventions, waitlist (WL), or usual care (UC) for CRF treatment. The outcomes included the Cancer Fatigue Scale (CFS) and Pittsburgh Sleep Quality Index (PSQI), and pair-wise and Bayesian network meta-analyses were performed using STATA v17.0.

**Results:**

In total, 34 randomized controlled trials featuring 2632 participants were included. In the network meta-analysis, the primary analysis using CFS illustrated that point application (PA) + UC (standardized mean difference [SMD] = −1.33, 95% CI = −2.02, −0.63) had the highest probability of improving CFS, followed by manual acupuncture (MA) + PA (SMD = −1.21, 95% CI = −2.05, −0.38) and MA + UC (SMD = −0.80, 95% CI = −1.50, −0.09). Moreover, the adverse events of these interventions were acceptable.

**Conclusion:**

This study demonstrated that acupuncture was effective and safe on CRF treatment. However, further studies are still warranted by incorporating more large-scale and high-quality RCTs.

**Systematic review registration:**

https://www.crd.york.ac.uk/PROSPERO, identifier CRD42022339769.

## Introduction

Cancer-related fatigue (CRF) is one of the most commonly reported symptoms impacting cancer survivors (1). CRF is estimated to occur in up to 90% of patients with cancer during active treatment and 27%–82% of patients after treatment (2). CRF is a common subjective symptom in patients with cancer that usually manifests as weakness, low endurance, and high energy consumption that cannot be relieved by sleep and rest (3). Differing from general fatigue, CRF is accompanied by a series of painful symptoms such as sleep disorders and mental fatigue that reduce patients’ tolerance, interfere with treatment, and seriously affect the quality of life and survival of patients. The incidence of insomnia in tumor-related diseases ranges from 18% to 68% (4, 5). Psychological pressure and negative emotions can easily cause fatigue symptoms, and the development of fatigue symptoms also aggravate the negative emotions of patients. Therefore, the management of CRF and its common comorbid symptoms (e.g., sleep disturbance) is an urgent issue during cancer treatment.

The etiology of CRF is multifactorial, and it consists of physiological, biochemical, and psychological factors that usually coexist and have additive effects. CRF can be attributed directly to cancer itself as well as its complications, the side effects of treatments, and patients’ comorbidities and psychosocial factors. CRF probably starts in the skeletal muscles because of a progressive reduction of physical activity (sometimes with physical interruption), but the brain is also critical as a central regulator of fatigue perception. A recent review found a positive correlation between fatigue and the circulating levels of inflammatory markers; in particular, interleukin (IL)-6, IL-1, and neopterin levels were significantly associated with CRF (1, 6, 7). The complexity of the etiological factors makes CRF difficult to treat. Conventional medical approaches can provide relief for some of the abovementioned symptoms, but often, solutions are limited (8, 9). The lack of proven effective treatments for CRF in conventional medical care leaves this common and distressful illness underreported and undertreated (10).

Acupuncture is widely used in palliative cancer care among Chinese populations. CRF is equivalent to the category of deficiency syndrome in traditional Chinese medicine. It is the main pathogenesis of the insufficiency of Zang-fu organs, qi and blood, Yin and Yang, and exhaustion after long deficiency (11). Numerous systematic reviews and meta-analyses (8, 12–14) suggest that acupuncture has clinical applications in the management of CRF. It was reported that acupuncture is commonly used to treat CRF to improve quality of life in patients with cancer (3, 15–25). However, are all types of acupuncture therapy efficacious in treating cancer-related symptoms and which acupuncture therapies provide the greatest efficacy? As an extension of pairwise meta-analysis of two treatments, network meta-analysis has recently attracted many researchers in evidence-based medicine because it simultaneously synthesizes both direct and indirect evidence from multiple treatments and thus facilitates better decision making. The Bayesian hierarchical model is a popular method to implement network meta-analysis, and it is generally considered more powerful than conventional pairwise meta-analysis, leading to more precise effect estimates with narrower credible intervals (26, 27). We conducted a network meta-analysis of randomized controlled trials (RCTs) (3, 16, 19–25, 28–52) to determine the efficacy of acupuncture therapies in CRF.

## Methods

The reporting of this Network Meta-analysis follows the Preferred Reporting Items for Network Meta-analysis (PRISMA) statement (53, 54).

## Protocol and registration

This network meta-analysis was registered on the PROSPERO platform (number: CRD42022339769) and reported following the Preferred Reporting Items for Systematic Reviews and Meta-Analysis checklist (55).

## Eligibility criteria and exclusion criteria

### Types of studies

All articles reporting RCTs published in English or Chinese were eligible without any regional and publication restrictions. The first period of the randomized cross-over trials was applied. Conversely, non-randomized clinical studies, quasi-RCTs, cluster RCTs, case reports, and studies for which no data were available were excluded.

### Types of participants

Trials enrolling adults with any type of cancer (≥18 years) were eligible. All cancer types were included because we assumed that CRF is a general cancer problem and that the working mechanisms and effects of the different interventions targeting CRF would be similar across all cancer diagnoses.

### Types of interventions

Acupuncture therapies were considered, including manual acupuncture (MA), electroacupuncture (EA), point application (PA), acupressure (AU), and transcutaneous electric acupoint stimulation (TEAS).

### Types of comparators

The control interventions included conventional treatment, usual care (UC), Chinese medicine (CM), sham acupuncture (SA), waitlist (WL), or no intervention, as well as other techniques. Laser acupuncture, acupoint injection, moxibustion, and transcutaneous electrical nerve stimulation were excluded.

### Types of outcome measurements

We included studies that covered at least one of the relevant outcomes. Our network meta-analysis primarily aimed to compare and rank the efficacy of acupuncture methods using the Cancer Fatigue Scale (CFS) and Pittsburgh Sleep Quality Index (PSQI). Using the CFS score, the primary acceptable outcomes were fatigue symptoms, as numerous previous studies reported the use of fatigue scales including the Revised Piper Fatigue Scale (56, 57), Piper Fatigue Scale, Brief Fatigue Inventory (56), and Karnofsky Performance Status (58). We divided fatigue into three levels: mild, 1–3 points; moderate, 4–6 points, and severe, 7–10 points (19). Regarding the secondary outcome, sleep quality was assessed using PSQI before and after the intervention. The 19-item PSQI instrument produces a global sleep quality score and seven specific component scores: quality, latency, duration, disturbance, habitual sleep efficiency, use of sleeping medications, and daytime dysfunction. Global scores range from 0 to 21, with higher scores indicating worse sleep quality and greater sleep disturbance (59).

## Search strategy

### Searching strategies

**Figure 1:**
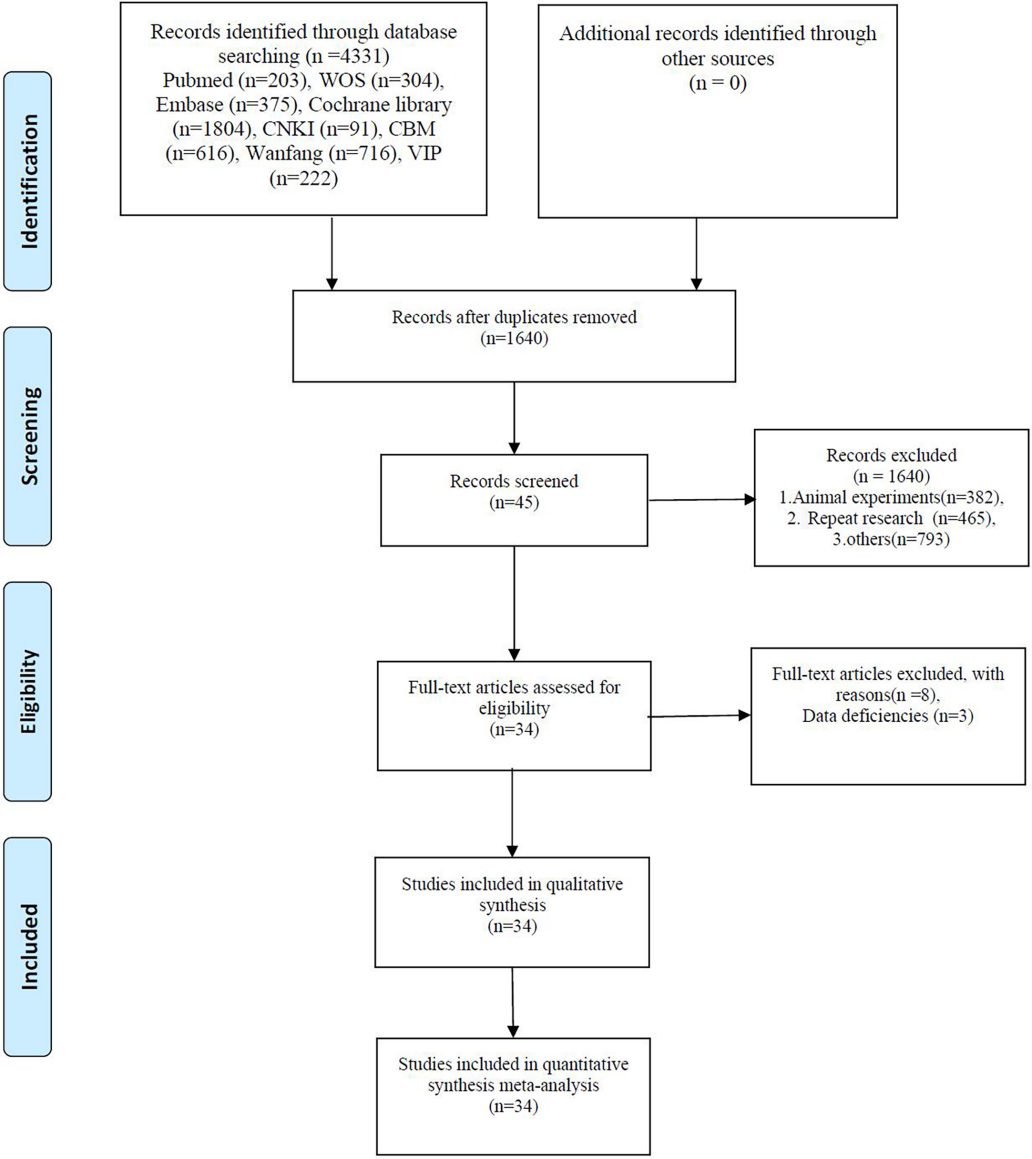
The PRISMA flow chart of selection process.

Eight databases, including PubMed, Embase, Web of Science, Cochrane Library, China Biology Medicine, China National Knowledge Infrastructure, and China Science and Technology Journal Database, were used (**Figure 1**). Databases were systematically searched from inception to November 2022 for correlative RCTs using the following keywords: “cancer related fatigue,” “cancer associated fatigue,” “acupuncture,” “electroacupuncture,” “point application,” “TEAS,” “transcutaneous electric acupoint stimulation,” “electroacupuncture,” “acupressure,” “randomized controlled trials,” and “RCT.” Two reviewers first screened the literature by scanning the titles and abstracts and then read the full text of potentially eligible trials to decide whether they should be included in the meta-analysis. The database search strategy is presented in **Appendix Supplementary Materials**.

### Study selection and data extraction

Data were extracted by two reviewers independently. Disagreements concerning data extraction were resolved by discussion. TH and YHC independently screened titles, abstracts, and keywords to identify duplicate trials and clearly ineligible studies for exclusion. Subsequently, the full text of the studies was examined to ensure that they met the inclusion criteria. Disagreements regarding study eligibility were resolved by a third reviewer.

The risk of bias of methodological quality was assessed using the Cochrane Risk of Bias (ROB2) Tool. This tool comprised six parts (randomization process, deviations from intended interventions, missing outcome data, measurement of the outcome, selection of the reported result, and overall bias) and ranked the methodological quality as some concerns, low risk, or high risk. A third party (ZW or FL) was consulted to assist in the final decision-making process. The ROB 2 plot was generated using the revised Cochrane risk of bias tool for randomized trials (RoB2).

### Data synthesis and statistical analysis

RevMan 5.3 was used to analyze the data. Pre–post differences were used as outcome indicators for each included study. Further, three-arm RCTs were separated into two arms for all possible combinations in the meta-analysis. The fixed-effects model utilized the Mantel–Haenszel procedure; otherwise, the random-effects model adopting the Der Simonian–Laird procedure was used. The I^2^ statistic and *p* value were used to identify and measure the heterogeneity among the studies. Low, moderate, and high heterogeneity were indicated by I^2^ values of <25%, 25%–50%, and >50%, respectively. *P* ≤ 0.1 was considered indicative of significant heterogeneity (60, 61). All data were analyzed with a 95% confidence interval (CI). We first performed pair-wise meta-analysis, followed by network meta-analysis. Pair-wise meta-analysis compared acupuncture treatment to the control. For continuous data, we calculated the standardized mean difference (SMD) or mean difference (MD) between treatments for CFS and PSQI scores. Cohen benchmarks for interpreting the magnitude of the SMD scores were used, where a result of ≤0.20 is considered a small effect, 0.50 is defined as moderate, and a large effect is >0.80 (62). A sensitivity analysis was performed to assess the robustness of the results in CFS and PSQI scores. This step repeated the primary analysis with a modified dataset based on assumptions of quality or study size. This led us to investigate whether these factors might have any effect on the main results. The sensitivity analysis was performed using STATA 17.0 software. The statistical method was used to assess the stability of results from direct comparisons to verify whether the NMA results were reliable.

### Publication bias

We generated a funnel plot to provide digital-based modeling of the results and eliminate bias (63).

### Quality of evidence

Using Grades of Recommendations, Assessment, Development, and Evaluation (GRADE) (64), the overall quality of evidence was assessed and ranked as high, moderate, low, or critically low.

## Results

### Selection of eligible studies

After the primary search process, we identified 4331 potentially relevant studies from these databases. After eliminating 465 duplicates as well as animal studies, reviews, and protocols, 45 articles were retained. Following a full-text assessment, 11 articles were excluded, and 34 RCTs were included in this systematic review (65).

### Characteristics of the included studies

Of the studies included in the final Bayesian meta-analysis, 18 RCTs (3, 15, 17, 21, 22, 24, 25, 32, 35–38, 42, 43, 47, 48, 52, 66) were published in Chinese, whereas 16 RCTs (16, 19, 23, 29–31, 34, 39–41, 44–46, 49–51) were written in English. The studies also included four three-arm (40, 42, 43, 48) and 29 two-arm studies. All 34 articles were published between 2006 and 2022, and they included a total of 2632 participants. Among the 34 trials, the mean age of the included patients ranged 43.12–69.73 years. Meanwhile, 33 studies provided details of the treatment acupoints, and all trials described the insertion technique. The interventions in these studies included MA, MA + UC, EA, AU + PA, PA, PA + UC, AU, TEAS, TEAS + UC, and other combinations with UC (including cancer-related drugs, CM, cognitive behavioral therapy, and waitlist [no intervention]). The detailed information of the included studies is presented in **Table 1**.

**Table 1:** Characteristics of included studies.

Study	country	sample size	Age (Year)	Gender(M:F)	Treatment group	Control group 1	Control group 2	Duration of treatment	Outcome	Course of diseases	Type of intervention	Outcome observation time	Adverse events
Cheng 2017	China	14/14	58±5.2/62±4.3	13:15	MA on LI4, Ren6, ST36, KI3, SP6	SA		Twice per week for 4 weeks	BFI/FACT	/	/	2w/4w/6w	No adverse events
SL Luo 2021	China	35/35	63.41±0.84/63.52±1.88	36/34	Ear acupressure Shenmen, Subcortex, Heart+conventional treatment	UC		Acupoints twice a day press once for 1 min, 5 days change to the other ear	Piper/treatment compliance score/QLQ-C30	1.52±0.42/1.75±0.51	Lung cancer	5d	/
Smith 2016	Australia	10/10/10	55±8.8/53±12.5/58±7.5		MA on KI3, KI27, ST36, SP6, CV4, CV6	SA	Wait list control were offered acupuncture after this time.	twice weekly for 3 weeks and then weekly for the final 3 weeks.	BFI		Breast cancer	2W/4W/6W	No adverse events
LJ Tao 2020	China	32/33	63.91 ± 7.92/61.30 ± 10.03	31/34	MA on ST36, SP6, PC6, RN4, LR4, KI3, HT7, GB12	UC		Once a day for 7 days	BFI/KPS/treatment compliance score	(22.1±10.4/21.9±10.5)m	Lung cancer:10, Liver cancer:3, Intestinal cancer:6, Ovarian cancer:5, Pancreatic cancer:1 and others	2w	/
XY Wang 2021	China	50/50	56.27±20.53/52.64±19.33	57/43	MA on Du20, Du26, KI1, LI4, SP6+ PA on RN22, PC6, KI1	UC		The application lasted for 5h, once a day	PFS	16.81±6.03/16.52±5.96	Esophageal cancer	2w、4w	/
Atefeh Ghanbari Khanghah 2019	Iran	30/30/30	PA (50.43±15.03) /S(51.83±12.8) /C(54.10±15.47)	53/37	AU on LI4, ST36, SP6	all points at a distance of 1.5 cm from the main acupoints were pressed	No intervention		VAS score of fatigue		GI, Breast, Leukemia, Lymphoma, MM Osteosarcoma, Lung, Ovary and Cervix, Ovary and Cervix	immediately	
Lu Lin 2019	China	34/32/34	59.50±9.21/61.85±8.48	41/27	Five ear PA on (the lung, Shen Men, subcortex, liver and spleen)	Control group in place of Shenmen Vaccariae	usual care	4-6 times per session, 5 sessions for 3 treatment	CFS/PSQI/SAS	/	lung cancer	9w	No adverse events
Suzanna M. 2016	USA	94/96			AU on DU20, Ren6, LI4, ST36, SP6, and KI3	HT7, PC6, and LV3	usual care	once daily for 6 weeks	BFI/PSQI/LQTQL		Breast cancer or skin cancer	3w/6w	Six adverse events in acupressure treatment
Chao Hsing Yeh 2015	USA	16/15	58.92±10.93	0:49	Ear AU on Shenmen, Sympathetic, Occiput, Subcortex nervous, Neurasthenia points, Corresponding points related to location of body pain (varied for each participant)	SA Ear AU		4 APA treatments once a week for 4 weeks.	BFI		Breast cancer or Skin cancer	4w/8w	21/31
Jun J. Mao 2013	USA	22/22/23	57.56+10.1/60.96+6.5)/60.6+8.2	4/67	EA group, at least 4 local points two pairs of electrodes were connected at the needles adjacent to the painful joint(s) with 2-hertz electrostimulation	SEA	10 real acupuncture treatments after 12 weeks	10 treatments for 8 weeks.	BFI/PSQI/HADS-Anxiety/HADS-Depression		Breast Cancer	4w/8w/12w	/
Denise Shuk Ting Cheung 2019	Hong Kong, China	15/15	61.8 ± 9.92/58.93 ± 11.11	6/24	AU on PC6, ST36, SP6, GV20, HT7, CV4, KI3, LV3, GB20, GB23, LI4	UC			BFI/PSQI/HADS-anxiety/HADS-depression/FACT	45.71 ± 46.7 /32.43 ± 19.23	Colorectal:7/lung:12/breast:10/prostate:1	4w/8w	No adverse events
Alexander 2006	UK	15/16/16(MA/PA/Sham)	53.4±13.1	0/47	MA+AU on ST36, SP6, LI4+Usual Care	UC		six sessions for 6 weeks.	MFI/HADS/FACT-B	Range:4-58/range: 2-69 months	Breast Cancer	6w	
Xiu-ting DU 2020	China	26/24	55.62 ±12.04/55.62 ±12.04	33/27	MA on ST36, CV4, CV6,	UC		1 day before chemotherapy, 1,2,3 of chemotherapy.8 treatment	PFS/EORTC-QLQ-C30		Intestinal cancer	3w/6w	No adverse events
Judith Balk 2009	USA	16/11	54(9.1)/53.7(9.0)	/	EA on KI3, SP6, LI4, ST36, Ren6, CV6. KI3 using low-frequency electrical stimulation (1 Hz)	SEA		once to twice per week for 6-week	FACT/SF-36		All subjects except one had breast cancer; the one remaining patient had endometrial cancer.	3w/6w/10W	No adverse events
Ülkü Özdemi 2021	Turkey	15/16	69.73 ± 4.72/70.37 ± 5.52	11/20	AU on LI4, SP6, ST36	WL		three minutes twice daily, for four weeks.	PFS	5.33±5.55/6.90±6.54	Breast Cancer:12, Colon Cancer:5,Lung Cancer:3,Lymphoma:6,Rectal Cancer:2,and others:3	4w	
XP Liu 2022	China	62/62/62	5.31 ~78(56.72 ±10. 30)	35:27/34:28/35:27	PA on ST36 +CM (Bazhen decoction)	CM	UC	14 days as a course of treatment, 2 course of treatment.	BFI/ QLQ-C30/ CD3^+^,CD4^+^, CD8^+^。	/	21 cases of small cell lung cancer, 18 cases of colon cancer and 23 cases of breast cancer	6w	11 serious adverse events
MT Zhu 2021	China	33/32	58.0 ±9.20/58.76 ±10.40	27/33	PA RN8+Ear AU Shenmen, spleen, stomach	UC		2 weeks as a course of treatment, 2 courses of treatment.	PFS/WBC/RBC/Hb/PLT/treatment compliance score	4.60 ± 2.90/3.60 ± 2.25	lung cancer:12/breast cancer:5/liver cancer:7/colon cancer:3 and others	8w	/
Y Li 2020	China	55/53	59.85±10.34/56.83±11.06	55/53	Treated with TEAS RN4, RN6, RN12, PC6, ST36, SP6	UC		20 minutes one time, once a day, for 5 days	PFS/FACT-G		lung cancer:28/breast cancer:7/ stomach cancer:15/colon cancer:40 and others	5d	/
LY Guo 2014	China	40/40/40	51 ± 5.5/52 ± 3.7/53 ± 6.2	0:120	MA in PC6, ST36, RN4, RN6	CM	No intervention	Once a day, 3 times a week, a total of 3 weeks.	PFS\treatment compliance score		Ovarian cancer:50/Cervical cancer:42/Endometrial cancer:28	3w	/
MW Yu 2017	China	36/36	50.2±8.0/51.4±8.4	0:72	MA in PC6, ST36, SP6, GV20, RN6	SA		Treatment was given twice a week for 4 weeks,	PFS/PSQI/HADS/ECOG/HADS		Breast cancer	4w/8w	/
Y Su 2016	China	30/30	60±11/62±6	32/28	MA on RN6, RN4, ST36, GB39, SP10, KI3	UC		Once a day treatment of 14 d.	KPS/WBC/lymphocyte/HB/PLT/treatment compliance score	/	lung cancer:19, Stomach cancer:10, Breast cancer:8, Pancreatic cancer:8, and others:16	14d	/
LL Yin 2021	China	54/54	43.12±5.14/44.19±4.98	55/53	MA + PA on ST36, SP6, KI1, EX-HN11, DU20, Du26+PA:KI1, RN8	UC		Once a day,4h, for 7d.	PFS/QOL	/	Esophageal Cancer	7d	
Deng 2013	USA	47/50	54/53	80/17	MA on CV6, CV4, KI3, ST36, SP6, LI11, HT6	SA		once weekly true or sham acupuncture for 6 weeks.	BFI, HADS, FACT	/	Breast Cancer, Cervical, CNS, Endometria and others	4w	/
YF Luan 2021		30/30	51.7 ±6.3/53.1 ±5.40:60		PA with CM in LR3, PC6, SP6, KI1	UC		For 10 d, one course of treatment was given every month for 3 consecutive courses of treatment to observe the curative effect.	PFS/PSQI		Breast cancer	3 consecutive courses of treatment.	
JX Gao 2022	China	41/41	50.26+4.1/50.02+4.1	64/18	AU on PC6, ST36, LR3, LR5,	UC			BFI/PSQI		Lung cancer	24h	
MZ Hu 2019	China	50/50	65. 24 ± 8.45/64.70 ± 9.65	64/36	EA on SP6, ST36, BL17, RN6, LI4			Acupuncture electrical stimulation was performed at 30 mim once per day on 13, 5, 7, 10, 14 and 21 days after chemotherapy, 7 times in total	PFS/SAS/SDS	/	Lung cancer	7d	
Ting Ma 2020	China	31/31	63.7±5.6/62.3±4.3	33/27	AU on RN12, PC6, ST36, HT7, with CM	UC		Each acupoint was massage 15 times, 3 times /d, replaced once for 24h and used for 14d,21d was a cycle, and observed for 3 cycles	PFS//CD4+/CD8+/Th1/Th2		Stomach cancer	63d	
Sheila N 2017	Canada	30/28	52.9±8.6/50.4±8.4	0/58	EA, the acupuncturist chose standard points	UC		A total of 10 treatments over 8 weeks	PSQI		Breast cancer	8w`	/
Melanie D 2021	Germany	26/26	56.58±7.9/54.8±8.3	0/52	the acupuncturist worked with a semi standardized protocol and placed the needles in sensitive points—first on the postantitragal belt, second on the helix channel, and finally on the HT7	UC		10 treatments within five weeks	PSQI/PSS/FACT-B/HADS-A/HADS-D	53.8±35.2/57.8±44.7(m)	Breast cancer	5w	At least twice included bruising, hunger pangs, flushing, heavy fatigue, tenderness, and pain
He SY 2021	China	40/40	56. 02 ± 10. 38	24/16	PA on HT7, PC6, ST36, SP6, RN8, BL15, BL20	UC		Once a day for a month	PSQI/Karnofsky	5. 33 ± 2. 15	Lung cancer	4w	/
Chi WC 2019	China	55/55	55.41±12.40/60.23±13.62	29/26	PA on HT7, KI3, SP6, BL62, KI6 with Tianwang Buxin Dan plus or minus prescription (CM)	UC		Acupuncture with traditional Chinese medicine for 14d	PSQI	/	Lung cancer	14d	The 19 patients with adverse reactions included drowsiness, dizziness, dry mouth, and ataxia
Zhang 2021	USA	15/15	52.5±8.9/52.7±6.3	0/30	Four pairs of electrodes from EA apparatus were connected to DU20 (+) and DU24 (−), center and right EX-HN1 (L+R−). For AA, V accaria seeds were embedded on surface of 3 auricular points (Heart, Shenmen, and Sympathetic) and maintained between 2 treatments.	WL		twice weekly for 6 weeks	PSQI/HADS/FACT-B/AES/ISI	/	Breast cancer	6w	5 events including skin allergic reaction, bruising and so on
Yoon 2019	Korea	23/23	45.05 ± 8.33/45.05 ± 8.33	0/46	Auricular therapy at the Shenmen, heart, anterior lobe, and occiput	SA group: (different area from the auricular points of the experiment group)		/	PSQI/ IL-6/ TNF-α/ CRP	/	Breast cancer	6W	/
Sheila N 2019	USA	80/80	62.3 ± 11.4/60.7 ± 12	69/91	Standardized points commonly used to address sleep problems with additional points to treat comorbid symptoms like pain and anxiety	CBT-I (included sleep restriction, stimulus control, cognitive restructuring, relaxation training, and education)		Acupuncture twice weekly for two weeks, then weekly for six more weeks, for a total of ten treatments over eight weeks	PSQI/ IL-6/ TNF-α/Cortisol/CRP		Breast/prostate/rectal/head/hematologic and other cancer	8w/20w	Acupuncture group reported nine event s ,control group 5 events

MA, manual acupuncture; EA, electroacupuncture; PA, point application; AU, acupressure; WL, Waitlist group; SA, Sham group; APA, Auricular point acupressure; UC, usual care; and PSQI, Pittsburgh Sleep Quality Index; KPS, Karnofsky Performance Status; BFI, Brief Fatigue Inventory (BFI-C); PFS, Piper Fatigue Scale; FACT, Functional Assessment of Cancer Therapy-Lung Cancer Subscale; EORTC-QLQ-C30, Quality of life; the European Organization for Research and Treatment of Cancer; ISI, Insomnia Severity Index; IL-6,interleukin-6; TNF-a, Tumor necrosis factor alpha; CRP, C-reactive protein; HADS, Hospital Anxiety and Depression Scale; FACT-B, Functional assessment; Gender, (M, Men; F, Female); Course of diseases, (Y, year; M, month; W, week; D, day).

### Risk of bias among the included RCTs

**Figure 2:**
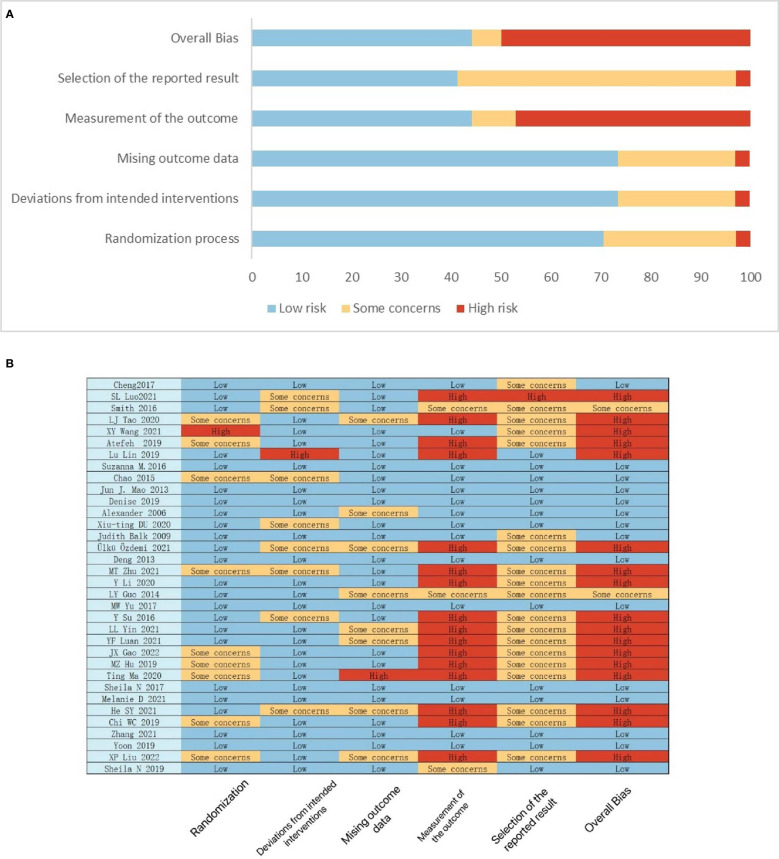
**(A)** Risk of bias graph; **(B)** Risk of bias summary.

The Cochrane ROB 2 Tool was used to assess the quality of all 34 trials included in our study. According to RoB 2, Two RCTs (40, 48) were rated as having some concerns regarding the risk of bias, and 17 RCTs (16, 20, 23, 24, 29, 31, 34, 37–39, 41, 44, 46, 47, 49–51) were rated as having a high risk of bias. Major sources of concerns include blind method leading to the outcome have been influenced by intervention, and a lack of prospectively developed analysis plans, which could have contributed to possible selection of reported results. The remaining 15 RCTs were rated as having a low risk of bias. SL Luo et al. (16) had a critical risk of measurement of the outcome due to outcome inappropriate. The detailed results of the risk of bias analyses are available in **Figure 2**.

### Results of the comparative effectiveness of the interventions

#### Pair-wise meta-analysis

Regarding the primary outcome, the meta-analysis revealed that the most commonly compared modalities were MA and SA (n = 5). The analysis found that MA + UC (SMD = −0.87, 95% CI = −1.19, −0.55; I^2^ = 0), PA + UC (SMD = −1.85, 95% CI = −3.19, −0.52; I^2^ = 94) and TEAS + UC (SMD = −1.04, 95% CI = −1.55, −0.54; I^2 =^ 0) were showed statistically effect to usual care, the quality of the evidence were low. AU (SMD = −0.66, 95% CI = −0.89, −0.43; I^2^ = 0), the quality of the evidence was moderate. PA + AU (SMD = −0.93, 95% CI = −1.53, −0.33) were superior to UC, the quality of the evidence was low. Meanwhile, MA (SMD = −0.64, 95% CI = −0.84, −0.44; I^2^ = 73%) and AU (SMD = −0.97, 95% CI = −1.70, −0.25; I^2^ = 0) displayed beneficial effects compared with SA, the quality of the evidence was moderate. AU (SMD = −1.97, 95% CI = −2.59, −0.91; I^2^ = 0) was beneficial compared with WL, the quality of the evidence was moderate. And EA (SMD = −1.90, 95% CI = −3.53, −0.27) were beneficial compared with WL, the quality of the evidence was low. Detailed results are presented in **Table 2**.

**Table 2:** Pairwise meta-analysis of CFS.

Comparison	Number	SMD (95%)	I² (%)	p
MA+UC VS UC	3	**-0.87[-1.19,-0.55]**	0	<0.0001
MA vs SA	5	**-0.64[-0.84,-0.44]**	**73**	<0.0001
MA vs WL	2	0.03[-0.28,0.33]	0	=0.86
PA+UC vs UC	3	**-1.85[-3.19,-0.52]**	94	=0.007
AU vs WL	2	**-1.97[-2.59,-0.91]**	0	<0.0001
AU vs UC	4	**-0.66[-0.89,-0.43]**	0	<0.0001
TEAS+UC vs UC	2	**-1.04[-1.55,-0.54]**	0	<0.0001
AU vs SA	3	**-0.97[-1.70,-0.25]**	0	=0.0008
EA vs SA	1	-0.80[-0.24,0.80]	/	0.12
EA vs WL	1	**-1.90[-3.53,-0.27]**	/	=0.02
AU+PA vs UC	1	**-0.93[-1.53,-0.33]**	/	=0.002
PA+CM vs UC	1	-0.48[-0.23,1.19]	/	0.61

MA, manual acupuncture; EA, electroacupuncture; PA, point application; TEAS, Transcutaneous Electric Acupoint Stimulation; AU, acupressure WL, Waitlist group; UC, usual care; SA, Sham group and CFS, Cancer Fatigue Scale.

Concerning PSQI, 13 studies compared different interventions. AU (SMD = −1.78, 95% CI = –3.06, −0.5; I^2^ = 89%), PA + UC (SMD = −2.13, 95% CI = −2.13, −1.43; I^2^ = 66), MA + CM (SMD = −2.58, 95% CI = −3.52, −1.64) produced significantly better outcomes than UC the quality of the evidence was critically low. And EA (SMD = −1.80, 95% CI = −3.16, −0.44), was showed significantly better outcomes than UC, the quality of the evidence was low. AU (SMD = −2.95, 95% CI, −4.07, −1.83) displayed better efficacy than SA, the quality of the evidence was moderate. AU + CM (SMD = −2.30, 95% CI, −3.51, −1.09) and AU (SMD = −2.70, 95% CI = −5.02, −0.38) produced significantly better outcomes than WL, the quality of the evidence was moderate. No significant differences were noted between EA and SC or between MA and WL. Detailed results are presented in **Table 3**.

**Table 3:** Pairwise meta-analysis of PSQI.

Comparison	Number	SMD (95%)	I² (%)	p
AU vs UC	4	**-1.78(-3.06,-0.5)**	89	=0.006
PA+UC vs UC	2	**-2.13(-2.13,-1.43)**	66	<0.0001
EA vs SA	1	-0.60(-2.42,1.22)	/	=0.65
AU vs SA	1	**-2.95(-4.07,-1.83)**	/	<0.0001
MA+CM vs UC	1	**-2.58[-3.52,-1.64]**	/	<0.0001
EA vs UC	1	**-1.80[-3.16, -0.44]**	/	<0.0001
AU+CM vs WL	1	**-2.30[-3.51,-1.09]**	/	P=0.00002
MA vs WL	1	**1.51[0.53,2.49]**	/	P=0.002
AU vs WL	1	**-2.70[-5.02,-0.38]**	/	P=0.02

MA, manual acupuncture; EA, electroacupuncture; PA, point application; TEAS, Transcutaneous Electric Acupoint Stimulation; AU, acupressure WL, Waitlist group; UC, usual care; SA, Sham group and PSQI, Pittsburgh Sleep Quality Index.

#### Network meta-analysis

**Figure 3:**
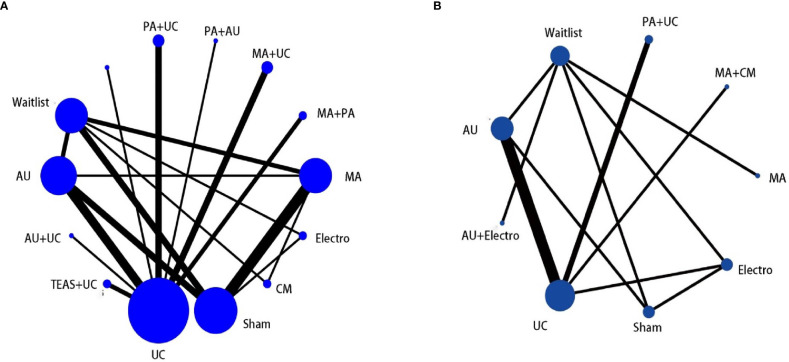
**(A)** The network graph of different interventions of CFS; **(B)** The network graph of different interventions of PSQI.

All 34 RCTs were included in this analysis. This study revealed that the most study comparison for CFS was acupuncture versus SA (n = 5), whereas that for PSQI was acupuncture versus UC (n = 4). The network plot is presented in **Figure 3**.

Concerning CSF, the network plot illustrated that the most common comparison was MA and SA (n = 5). The analysis found that PA + UC (SMD = −1.33, 95% CI = −2.02, −0.63), MA + PA (SMD = −1.21, 95% CI = −2.05, −0.38) and MA + UC (SMD = −0.80, 95% CI = −1.50, −0.09) were superior to UC. PA + UC (SMD = −1.46, 95% CI, −2.59, −0.33) and MA + PA (SMD = −1.35, 95% CI, −2.56, −0.13) displayed beneficial effects compared with SA. PA + UC (SMD = −1.67, 95% CI = −2.82, −0.51), MA + PA (SMD = −1.55, 95% CI = −2.80, −0.31), AU (SMD = −0.82, 95% CI, −1.52, −0.11), and MA (SMD = −0.67, 95% CI = −1.33, −0.01) were more beneficial than WL **Table 4**.

**Table 4:** The results of network meta-analysis of CFS (appendix).

PA+UC												
-0.11 (-1.20,0.97)	MA+PA											
-0.53 (-1.52,0.46)	-0.42 (-1.51,0.67)	MA+UC										
-0.55 (-1.95,0.84)	-0.44 (-1.91,1.03)	-0.02(-1.42,1.38)	PA+AU									
-0.85 (-1.77,0.07)	-0.74 (-1.76,0.29)	-0.32 (-1.24,0.61)	-0.30 (-1.65,1.06)	AU								
-0.88 (-1.96,0.19)	-0.77 (-1.94,0.40)	-0.35 (-1.43,0.73)	-0.33 (-1.80,1.13)	-0.03 (-1.05,0.98)	TEASS+UC							
-1.00 (-2.18,0.19)	-0.88 (-2.15,0.39)	-0.46 (-1.66,0.73)	-0.44 (-1.99,1.10)	-0.15 (-0.90,0.61)	-0.11 (-1.38,1.15)	MA						
-1.06 (-2.61,0.48)	-0.95 (-2.56,0.66)	-0.53 (-2.08,1.01)	-0.51 (-2.35,1.32)	-0.21 (-1.45,1.03)	-0.18 (-1.78,1.42)	-0.07 (-1.27,1.13)	electro					
**-1.33 (-2.02,-0.63)**	**-1.21 (-2.05,-0.38)**	**-0.80 (-1.50,-0.09)**	-0.77 (-1.99,0.44)	-0.48 (-1.08,0.12)	-0.44 (-1.26,0.38)	-0.33 (-1.29,0.63)	-0.26 (-1.64,1.11)	UC				
**-1.46 (-2.59,-0.33)**	**-1.35 (-2.56,-0.13)**	-0.93 (-2.06,0.20)	-0.91 (-2.41,0.60)	-0.61 (-1.26,0.05)	-0.58 (-1.79,0.63)	-0.46 (-1.00,0.07)	-0.40 (-1.52,0.72)	-0.13 (-1.02,0.76)	sham			
**-1.66 (-3.06,-0.27)**	**-1.55 (-3.02,-0.09)**	-1.13 (-2.53,0.26)	-1.11 (-2.82,0.60)	-0.81 (-2.16,0.53)	-0.78 (-2.24,0.68)	-0.67 (-2.21,0.87)	-0.60 (-2.43,1.23)	-0.34 (-1.54,0.87)	-0.21 (-1.70,1.29)	PA+CM		
**-1.67 (-2.82,-0.51)**	**-1.55 (-2.80,-0.31)**	-1.14 (-2.30,0.03)	-1.11 (-2.64,0.41)	**-0.82 (-1.52,-0.11)**	-0.78 (-2.02,0.46)	**-0.67 (-1.33,-0.01)**	-0.60 (-1.72,0.52)	-0.34 (-1.27,0.59)	-0.21 (-0.81,0.39)	-0.00 (-1.52,1.52)	Waitlist	
**-4.40 (-5.97,-2.83)**	**-4.29 (-5.92,-2.65)**	**-3.87 (-5.44,-2.30)**	**-3.85 (-5.71,-1.99)**	**-3.55 (-4.82,-2.28)**	**-3.52 (-5.15,-1.89)**	**-3.41 (-4.55,-2.26)**	**-3.34 (-4.90,-1.78)**	**-3.07 (-4.48,-1.67)**	**-2.94 (-4.13,-1.75)**	**-2.74 (-4.59,-0.88)**	**-2.74 (-3.88,-1.59)**	CM

Significant difference; MA, manual acupuncture; EA, electroacupuncture; PA, point application; TEAS, Transcutaneous Electric Acupoint Stimulation; AU, acupressure WL, Waitlist group; UC, usual care; SA, Sham group and Cancer Fatigue Scale.

Concerning PSQI, AU was more effective than UC (MD = −1.50, 95% CI = −2.90, −0.09) and WL (MD = −2.63, 95% CI = −5.09, −0.17). Further analysis revealed that PA + UC (MD = −5.33, 95% CI = −10.11, −0.55), MA + CM (MD = −5.19, 95% CI = −9.93, −0.45), and AU (MD = −4.11, 95% CI = −7.82, −0.40) were more beneficial than MA **Table 5**.

**Table 5:** The results of network meta-analysis of PSQI (appendix).

PA+UC								
-0.14 (-4.14,3.85)	MA+CM							
-1.05 (-4.94,2.85)	-0.90 (-4.75,2.94)	PA						
-1.22 (-4.41,1.96)	-1.08 (-4.21,2.04)	-0.18 (-3.18,2.82)	AU					
-1.79 (-5.49,1.92)	-1.65 (-5.29,2.00)	-0.74 (-4.28,2.80)	-0.56 (-2.99,1.87)	Electroacupuncture				
-2.72 (-5.58,0.14)	-2.58 (-5.37,0.21)	-1.67 (-4.32,0.98)	**-1.50 (-2.90,-0.09)**	-0.93 (-3.29,1.42)	UC			
-3.39 (-7.17,0.39)	-3.25 (-6.97,0.48)	-2.34 (-5.96,1.28)	-2.16 (-4.40,0.08)	-1.60 (-4.18,0.98)	-0.67 (-3.14,1.80)	sham		
-3.85 (-7.75,0.05)	-3.71 (-7.56,0.14)	-2.81 (-6.55,0.94)	**-2.63 (-5.09,-0.17)**	-2.07 (-4.74,0.61)	-1.13 (-3.78,1.52)	-0.46 (-3.00,2.07)	Waitlist	
**-5.33 (-10.11,-0.55)**	**-5.19 (-9.93,-0.45)**	-4.29 (-8.94,0.37)	**-4.11 (-7.82,-0.40)**	-3.55 (-7.40,0.31)	-2.61 (-6.45,1.22)	-1.95 (-5.71,1.82)	-1.48 (-4.27,1.31)	MA

MA, manual acupuncture; EA, electroacupuncture; PA, point application; AU, acupressure WL, Waitlist group; CM, Chinese medicine; UC, usual care; SA, Sham group; PSQI, Pittsburgh Sleep Quality Index.

#### Rank probabilities of the interventions

**Figure 4:**
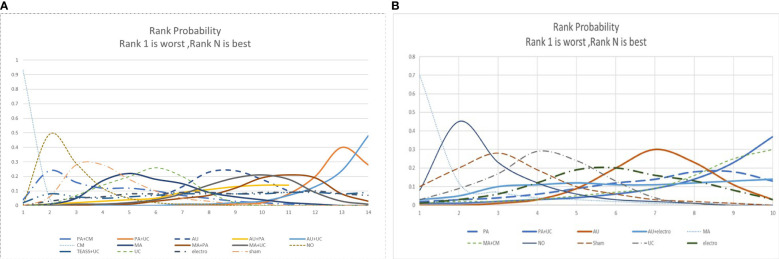
**(A)**The figure of ranking probability of reduction in CFS; **(B)** The figure of ranking probability of reduction PSQI.

We performed network meta-analysis using the consistency model, and a ranking probability figure was generated for CFS. Based on the figure, AU + UC, PA + UC, and MA + PA were the three most effective treatment modalities in the analysis. The cumulative SUCRA represents the probability that each intervention ameliorates the symptoms of CRF. This analysis revealed that AU + UC (100%) exhibited the highest probability of relieving CRF symptoms, followed by PA + UC (84.4%), MA + PA (80.6%), MA + UC (66.9%), PA + AU (64.2%), AU (56.0%), TEAS + UC (52.3%), MA (49.7%), EA (46.0%), UC (30.6%), SA (26.4%), PA + CM (23.6%), WL (18.6%), and CM (0.0%) **Figure 4A**.

We performed Bayesian analysis *via* the consistency model, and a ranking probability figure was generated for PSQI. PA + UC, AU, and EA were the three most effective methods in the analysis. Based on the cumulative SUCRA, PA + AU (83.1%) displayed the highest probability of improving PSQI, followed by AU (67.2%), EA (56.6%), UC (34.7%), SA (25.3%), WL (18.5%), and MA (5.9%) **Figure 4B**.

### Safety

Notably, only 13 RCTs described safety data. The therapies included MA, PA, and EA, PA caused bruising and minor comfort skin trauma. Acupuncture caused pain, headache, heavy eyelids, fatigue, and tenderness. These adverse events were acceptable. However, serious events including bronchospasm, low blood counts, renal failure nausea, vomiting, and small bowel obstruction also existed and required additional attention **Table 1**.

### Sensitivity analysis

**Figure 5:**
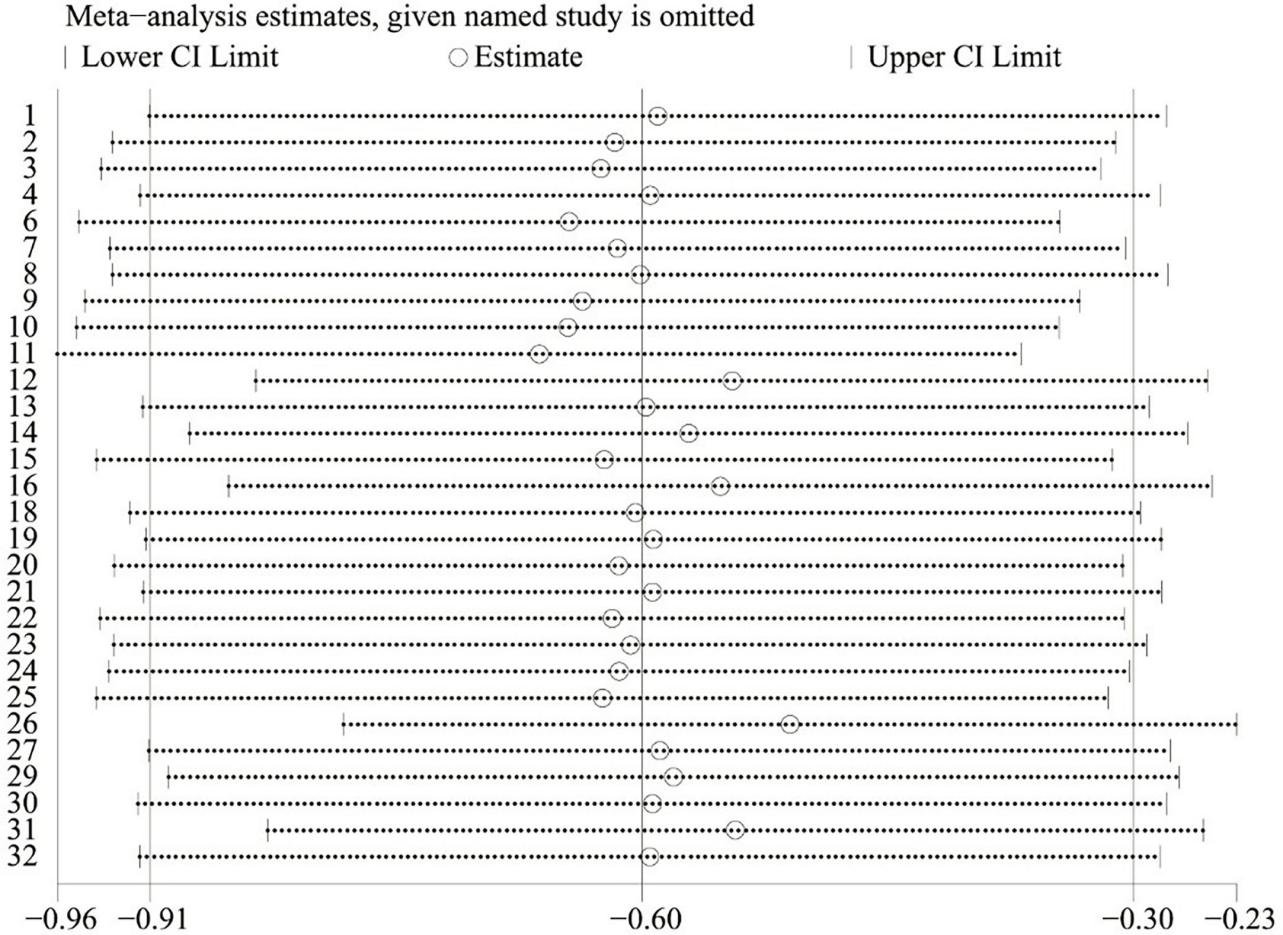
Sensitivity analysis for the network meta-analysis of CFS.

Sensitivity analyses confirmed the robustness of the results of all included studies in the fatigue scores, and results were less likely to be biased and unreliable **Figure 5**.

### Cluster ranking plot of different treatments

**Figure 6:**
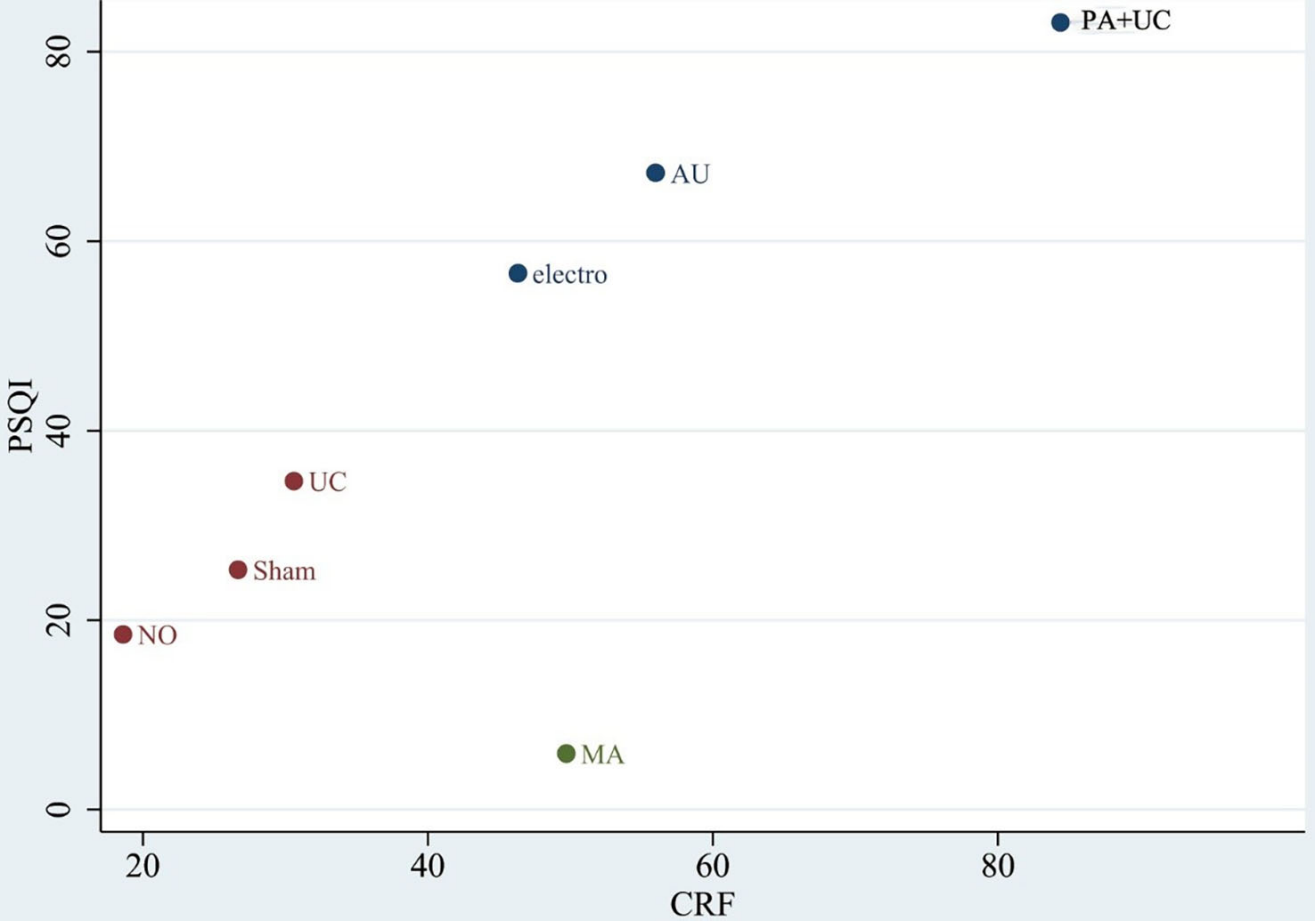
Clustered ranking plot. The plot is based on cluster analysis of surface under the cumulative ranking curves (SUCRA) values. Each plot shows the SUCRA values for two outcomes CFS: Cancer Fatigue Scale (CFS) and Pittsburgh Sleep Quality Index (PSQI).

The clustered ranking plot was based on cluster analysis of SUCRA for CFS and PSQI. Seven interventions with data for CFS and PSQI were analyzed. The results illustrated that PA + UC was most effective in improving both CFS and PSQI **Figure 6**.

### Publication bias

**Figure 7:**
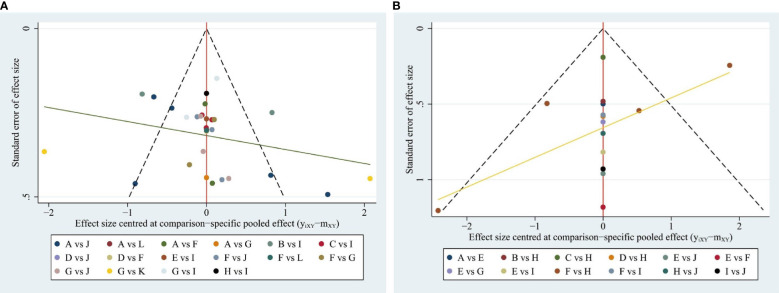
**(A)** Funnel plot for the network meta-analysis in CFS; **(B)** Funnel plot for the network meta-analysis in PSQI.

Publication bias was evaluated by comparing the symmetry of the funnel plot. As presented in **Figure 7**. The figure demonstrated a decreased likelihood of small sample effects. However, there was no strong and powerful evidence of these small study effects across the outcomes. To determine whether potential publication bias existed in the reviewed literature, Egger’s test was also carried out. The results of Egger’s test (P = 0.910) demonstrated no small-study effects and bias concerning fatigue scores (67, 68).

### Quality of evidence

According to the GRADE tool, the quality of the two outcomes (CFS and PSQI) was moderately to critically low. Because of risks of bias, inconsistency, and imprecision, most evidence was rated low **Tables 6**, **7**.

**Table 6:** Quality of evidence of CFS in network meta-analysis.

Outcome	Number of studies	Risk of bias	Inconsistency	Indirectness	Imprecision	Other Considerations	Acupuncture group	Control Group	Absolute	Certainty
MA+UC vs UC	3	Not Serious	Not Serious	Not Serious	Not Serious	Unclear	88	87	-0.87[-1.19,-0.55],I^2^=0, P=0.0001	⊕⊕⊕⊕ (moderate))
MA vs Sham	5	Not serious	Serious	Not serious	Not serious	Unclear	119	123	-0.64[-0.84,-0.44],I^2^=73 P=0.0001	⊕⊕⊕○ (low)
MA vs Waitlist	2	Not serious	Not Serious	Not serious	Serious	Unclear	49	50	0.03[-0.28,0.33],I^2^=0 P=0.86	⊕⊕⊕○ (low)
PA+UC vs UC	3	Not serious	Serious	Not Serious	Not serious	Unclear	124	124	-1.85[-3.19,-0.52],I^2^=94% P==0.007	⊕⊕⊕○ (low)
AU vs Waitlist	2	Not serious	Not serious	Not serious	Not serious	Unclear	45	46	-1.97[-2.59,-0.91], I^2^=0, P=0.0001	⊕⊕⊕⊕ (moderate)
AU vs UC	4	Not serious	Not serious	Not serious	Not serious	Unclear	178	180	-0.66[-0.89,-0.43] , I^2^=0, P=0.0001	⊕⊕⊕⊕ (moderate)
AU vs sham	3	Not serious	Not serious	Not serious	Not serious	Unclear	55	58	-0.97[-1.70,-0.25] , I^2^=0, P=0.0001	⊕⊕⊕⊕ (moderate)
Electroacupuncture vs sham	1	Not serious	Not serious	Not serious	Serious	Unclear	8	10	-0.80[-0.24,0.80] P=0.12	⊕⊕⊕○ (low)
Electroacupuncture vs Waitlist	1	Not serious	Not serious	Not serious	Serious	Unclear	8	10	-1.90[-3.53,-0.27] P=0.02	⊕⊕⊕○ (low)
TEAS+UC vs UC	2	Not serious	Not serious	Not serious	Not serious	Unclear	55	53	-1.04[-1.55,-0.54] I^2^=0, P=0.0001	⊕⊕⊕○ (low)
AU+PA vs UC	1	Unclear	Not serious	Not serious	Not serious	Unclear	30	30	-1.90[-3.53,-0.27] P=0.02	⊕⊕⊕○ (low)
PA+CM vs UC	1	Not serious	Not serious	Unclear	Serious	Unclear	30	30	-0.48[-0.23,1.19], P=0.61	⊕⊕○ (Critically Low)

**Table 7:** Quality of evidence of PSQI in network meta-analysis.

Outcome	Number of studies	Risk of bias	Inconsistency	Indirectness	Imprecision		Other Considerations		Acupuncture group		Control Group	Effect Relative Absolute		Certainty	
AU+CM vs Waitlist	1	Not Serious	Not Serious	Not Serious	Not Serious		Unclear		30		28	-2.30[-3.51,-1.09], P=0.00002		⊕⊕⊕⊕ (moderate))	
MA vs Waitlist	1	Not serious	Not Serious	Not serious	Serious		Unclear		80		80	1.51[0.53,2.49], P=0.02		⊕⊕⊕○ (low)	
PA+UC vs UC	2	Not serious	Serious	Not Serious	Serious		Unclear		85		85	-2.13(-2.13,-1.43)],I^2^=66% P==0.00001		⊕⊕○○ (Critically Low)	
AU vs Waitlist	1	Not serious	Not serious	Not serious	Not serious		Unclear		45		46	-2.70[-5.02,-0.38], P=0.002		⊕⊕⊕⊕ (moderate)	
AU vs UC	4	Not serious	Serious	Not serious	Not serious		Unclear		191		197	-1.78(-3.06,-0.5) , I^2^=89, P=0.006		⊕⊕⊕○ (low)	
AU vs sham	1	Not serious	Not serious	Not serious	Not serious		Unclear		41		41	-2.95(-4.07,-1.83) , P=0.0001		⊕⊕⊕⊕ (moderate)	
MA+CM vs UC	1	serious	Unclear serious	Not serious	Not Serious		Unclear		55		55	-2.58[-3.52,-1.64] P<0.0001		⊕⊕○○ (Critically Low)	
EA vs UC	1	Unclear	Not serious	Not serious	Not serious		Unclear		22		22	-1.80[-3.16,-0.44] P=<0.0001		⊕⊕⊕○ (low)	
PA+CM vs UC	1	Not serious	Not serious	Unclear	Serious		Unclear		30		30	-0.48[-0.23,1.19], P=0.61		⊕⊕○ (Critically Low)	

## Discussion

As it includes physical, mental, and emotional aspects, CRF is a multidimensional illness, and it can significantly affect patients’ lives. Approximately one-third of women experience moderate-to-severe persistent fatigue up to 10 years after the end of cancer treatment (69, 70). Additionally, persistent fatigue is associated with higher rates of poor sleep (70), and approximately 40%–70% of breast cancer survivors report sleep problems years after their diagnoses (71–73). These studies provided a mechanistic basis for the efficacy of acupuncture against CRF. Numerous studies found that the symptoms of fatigue (18, 42, 43, 47) and poor sleep (32, 52, 66) were alleviated by acupuncture. However, acupuncture therapies are complex, requiring significant manpower and high expenditures. Therefore, this network meta-analysis aimed to identify the optimal acupuncture therapy for patients with cancer using the most comprehensive information.

Recently, complementary approaches such as acupuncture have been increasingly scrutinized as alternate strategies for cancer-related symptom management. Acupuncture is recommended by National Comprehensive Cancer Network guidelines, especially for patients who have finished anticancer treatment and are defined as cancer survivors. Its efficacy and safety have been tested in a number of RCTs (74–76). Among the 34 trials included in this study, ST36 (stomach meridian), KI3, SP6 (convergent acupoint of the liver, spleen, and kidney Yin channels), and energy-associated points (77) including LI4 and DU20 can be considered the core acupoint combinations for treating CRF. In traditional Chinese medicine theory, blockages in the body’s life energy (Qi) are believed to be responsible for illness and disease (14). One study (78) found that acupuncture might alleviate fatigue by influencing neuter regulation, endocrine regulation, immune regulation, rhythm regulation, and other aspects of cancer fatigue (79).

This meta-analysis of the efficacy of different acupuncture treatments for CRF yielded reliable results. First, in the pair-wise meta-analysis, EA, AU displayed significantly better efficacy in improving CRF than WL. In addition, MA + UC, PA + UC, AU, TEAS + UC, and AU + PA had better efficacy than UC. Meanwhile, concerning sleep quality, AU, PA + UC, MA + CM, and EA displayed better efficacy than UC. Moreover, the network Bayesian meta-analysis indicated that PA + UC was a more optimal treatment than CM and MA for improving CFS and PSQI scores. Moreover, 13 trials reported the adverse events. Six studies recorded no adverse events. One study (22) reported serious events (including bronchospasm, low blood counts, and renal failure). Six studies reported mild acupuncture-related adverse events (e.g., pain, bruising, tenderness). In addition, this network meta-analysis had several strengths. To the best of our knowledge, it was the first network meta-analysis to evaluate and compare several different acupuncture therapies for the treatment of CRF. The SUCRA were utilized in this study to evaluate changes in CRF and sleep quality following various acupuncture treatments.

However, the study also included some limitations. First, the search language was limited to Chinese and English, which might inevitably cause bias. Second, some interventions included were poorly studied, which might lead to insufficient statistical efficiency. Third, we evaluated which acupuncture procedures would be the best alternative to conventional treatment, and the types of Western drugs and routine rehabilitation exercise were not studied separately. This was mainly because of the multitude of such treatments and their applications. These issues will be the focus of future studies. Fourth, the GRADE tool determined that the overall quality of evidence obtained from included studies was low. Fifth, considerable heterogeneity was noted in certain outcomes of pair-wise meta-analysis and might be attributed to the clinical, statistical, and methodological differences. Subgroup analysis was infeasible due to the insufficient number of studies included, and sensitivity analysis confirmed the reliability of the results.

Given that the reported outcomes of this study were subjective symptom scores, blinding of outcomes evaluators should be applied in RCTs to reduce bias and increase the reliability of the results. The RCT methodology of the included studies was generally poor because most of the risk items were unclear, particularly allocation concealment and blinding. In the future, we recommend researchers follow the CONSORT reporting statement to improve reporting quality. In addition, we recommend that the protocols should be registered.

## Conclusion

This Bayesian network meta-analysis demonstrated that acupuncture was an effective and safe strategy for alleviating cancer-related fatigue. However, due to the undesirable heterogeneity and quality of the included RCTs, the results should be interpreted with caution. Further studies are warranted by incorporating more large-scale and high-quality RCTs.

## Data availability statement

The original contributions presented in the study are included in the article/**Supplementary Material**. Further inquiries can be directed to the corresponding author.

## Author contributions

HT and YHC designed the search strategy. GXX, MSS and CYY performed the literature search. HT and LYH screened the studies for eligibility and wrote the first draft the manuscript. CYY, QL, and LYH performed the data extractions. HT, LYH, CYY conducted the statistical analyses. ZW, LZ and FRL were responsible for the manuscript editing and review of the manuscript. All authors contributed to the article and approved the submitted version.

## References

[1] Bower JE . Cancer-related fatigue–mechanisms, risk factors, and treatments. Nat Rev Clin Oncol (2014) 11(10):597–609. doi: 10.1038/nrclinonc.2014.127 25113839 PMC4664449

[2] Kirshbaum M . Cancer-related fatigue: a review of nursing interventions. Br J Community Nurs (2010) 15(5):214–6, 218-9. doi: 10.12968/bjcn.2010.15.5.47945 20453821

[3] Zick SM Sen A Wyatt GK Murphy SL Arnedt JT Harris RE . Investigation of 2 types of self-administered acupressure for persistent cancer-related fatigue in breast cancer survivors: A randomized clinical trial. JAMA Oncol (2016) 2(11):1470–6. doi: 10.1001/jamaoncol.2016.1867 27388752

[4] Li J Zhu C Liu C Su Y Peng X Hu X . Effectiveness of eHealth interventions for cancer-related pain, fatigue, and sleep disorders in cancer survivors: A systematic review and meta-analysis of randomized controlled trials. J Nurs Scholarsh (2022) 54(2):184–90. doi: 10.1111/jnu.12729 34791779

[5] Zhou ES Partridge AH Syrjala KL Michaud AL Recklitis CJ . Evaluation and treatment of insomnia in adult cancer survivorship programs. J Cancer Surviv (2017) 11(1):74–9. doi: 10.1007/s11764-016-0564-1 PMC5865603 27495283

[6] Morrow GR . Cancer-related fatigue: causes, consequences, and management. Oncologist (2007) 12 Suppl 1:1–3. doi: 10.1634/theoncologist.12-S1-1 17573450

[7] Ryan JL Carroll JK Ryan EP Mustian KM Fiscella K Morrow GR . Mechanisms of cancer-related fatigue. Oncologist (2007) 12 Suppl 1:22–34. doi: 10.1634/theoncologist.12-S1-22 17573453

[8] Finnegan-John J Molassiotis A Richardson A Ream E . A systematic review of complementary and alternative medicine interventions for the management of cancer-related fatigue. Integr Cancer Ther (2013) 12(4):276–90. doi: 10.1177/1534735413485816 23632236

[9] Mustian KM Alfano CM Heckler C Kleckner AS Kleckner IR Leach CR . Comparison of pharmaceutical, psychological, and exercise treatments for cancer-related fatigue: A meta-analysis. JAMA Oncol (2017) 3(7):961–8. doi: 10.1001/jamaoncol.2016.6914 PMC5557289 28253393

[10] David A Hausner D Frenkel M . Cancer-related fatigue-is there a role for complementary and integrative medicine? Curr Oncol Rep (2021) 23(12):145. doi: 10.1007/s11912-021-01135-6 34743258

[11] Wang L Xie Z Wu X Lv Q . Clinical observation of TCM syndrome differentiation on treatment of cancer-related fatigue in patients with breast cancer. China J Traditional Chin Med Pharm (2016) 12(31):5375–8.

[12] Jang A Brown C Lamoury G Morgia M Boyle F Marr I . The effects of acupuncture on cancer-related fatigue: Updated systematic review and meta-analysis. Integr Cancer Ther (2020) 19:1534735420949679. doi: 10.1177/1534735420949679 32996339 PMC7533944

[13] Posadzki P Moon TW Choi TY Park TY Lee MS Ernst E . Acupuncture for cancer-related fatigue: A systematic review of randomized clinical trials. Support Care Canc (2013) 21(7):2067–73. doi: 10.1007/s00520-013-1765-z 23435597

[14] Tao WW Jiang H Tao XM Jiang P Sha LY Sun XC . Effects of acupuncture, tuina, tai chi, qigong, and traditional Chinese medicine five-element music therapy on symptom management and quality of life for cancer patients: A meta-analysis. J Pain Symptom Manage (2016) 51(4):728–47. doi: 10.1016/j.jpainsymman.2015.11.027 26880252

[15] Cheng CS Chen LY Ning ZY Zhang CY Chen H Chen Z . Acupuncture for cancer-related fatigue in lung cancer patients: a randomized, double blind, placebo-controlled pilot trial. Supportive Care Cancer: Off J Multinat Assoc Supportive Care Canc (2017) 25(12):3807–14. doi: 10.1007/s00520-017-3812-7 28707168

[16] Luo S Liu Q Wen R Ge Y . Effect of evidence-based nursing combined with auricular point and finger pressure on cancer-related fatigue in patients with lung cancer undergoing radiotherapy. Chin Med Modern Distance Educ of China (2021) 19(24):145–7. doi: 10.3969/j.issn.1672-2779.2021.24.055

[17] Cheung DST Yeung WF Chau PH Lam TC Yang M Lai K . Patient-centred, self-administered acupressure for Chinese advanced cancer patients experiencing fatigue and co-occurring symptoms: A pilot randomised controlled trial. Eur J Cancer Care (Engl) (2020) 00:e13314. doi: 10.1111/ecc.13314 32896014

[18] Molassiotis A Bardy J Finnegan-John J Mackereth P Ryder WD Filshie J . A randomized, controlled trial of acupuncture self-needling as maintenance therapy for cancer-related fatigue after therapist-delivered acupuncture. Ann Oncol (2013) 24(6):1645–52. doi: 10.1093/annonc/mdt034 23436910

[19] Du XT Tian W Liu B Li . Prevention and treatment of acupuncture for cancer-related fatigue caused by chemotherapy of intestinal cancer: A randomize d controlle d trial. World J Acupuncture-Moxibustion (2021) 31(02):83–8.

[20] Liu X Li X Chen X Gao W . Bazhen decoction combined with acupoint application in the treatment of cancer-related fatigue. Chin J Clin Oncol Rehabil (2022) 29(03):328–32. doi: 10.13455/j.cnki.cjcor.2022.03.18.

[21] Yeh CH Chien LC Lin WC Bovbjerg DH van Londen GJ . Pilot randomized controlled trial of auricular point acupressure to manage symptom clusters of pain, fatigue, and disturbed sleep in breast cancer patients. Cancer Nursing (2016) 39(5):402–10. doi: 10.1097/ncc.0000000000000303 26390073

[22] Deng G Chan Y Sjoberg D Vickers A Yeung KS Kris M . Acupuncture for the treatment of post-chemotherapy chronic fatigue: a randomized, blinded, sham-controlled trial. Support Care Canc (2013) 21(6):1735–41. doi: 10.1007/s00520-013-1720-z PMC3953893 23334562

[23] Yin L Chen J Qi Y Zhang R . Effect of acupuncture combined with acupoint application on swallowing function and cancer-related fatigue of advanced esophageal cancer patients. J Esophageal Dis (2021) 3(03):218–21. doi: 10.15926/j.cnki.issn2096-7381.2021.03.014

[24] Li Y Zhang H Liu Z Hu L Liu H Yuan L . Clinical effect of transcutaneous electric acupoint stimulation in the treatment of cancer-related fatigue after chemotherapy. China Med Herald (2020) 17(12):149–68.

[25] Zhang J Qin Z So TH Chen H Lam WL Yam LL . Electroacupuncture plus auricular acupressure for chemotherapy-associated insomnia in breast cancer patients: A pilot randomized controlled trial. Integr Cancer Ther (2021) 20:15347354211019103. doi: 10.1177/15347354211019103 34036813 PMC8161840

[26] Lin L Chu H Hodges JS . On evidence cycles in network meta-analysis. Stat Interface (2020) 13(4):425–36. doi: 10.4310/sii.2020.v13.n4.a1 PMC7394478 32742550

[27] Rucker G Petropoulou M Schwarzer G . Network meta-analysis of multicomponent interventions. Biom J (2020) 62(3):808–21. doi: 10.1002/bimj.201800167 PMC7217213 31021449

[28] Cheung DST Yeung WF Chau PH Lam TC Yang M Lai K . Patient-centred, self-administered acupressure for Chinese advanced cancer patients experiencing fatigue and co-occurring symptoms: A pilot randomised controlled trial. Eur J Cancer Care (Engl) (2022) 31(5):e13314. doi: 10.1111/ecc.13314 32896014

[29] Chi W Wang B Yang F Jiang Q Jiang J . Clinical effect of acupuncture combined with medicine in the treatment of insomnia caused by cancer in patients with non-small cell lung cancer after chemotherapy. China Med Herald (2019) 16(23):114–7.

[30] Huang F Zhao Y . Transcutaneous electrical acupoint stimulation for cancer-related fatigue : Randomized controlled trial. Chin J Rehabil Med (2019) 34(06):688–92.

[31] Gao J Zhang L . Influence of TCM emotional nursing combined with acupoint massage on sleep quality and cancer-related fatigue in chemotherapy patients with lung cancer. Clin Med Engineering (2022) 29(01):125–6. doi: 10.3969/j.issn.1674-4659.2022.01.0125

[32] Garland SN Xie SX Li Q Seluzicki C Basal C Mao JJ . Comparative effectiveness of electro-acupuncture versus gabapentin for sleep disturbances in breast cancer survivors with hot flashes: a randomized trial. Menopause (2017) 24(5):517–23. doi: 10.1097/GME.0000000000000779 PMC5403590 27875389

[33] Halpin DMG Criner GJ Papi A Singh D Anzueto A Martinez FJ . Global initiative for the diagnosis, management, and prevention of chronic obstructive lung disease. the 2020 GOLD science committee report on COVID-19 and chronic obstructive pulmonary disease. Am J Respir Crit Care Med (2021) 203(1):24–36. doi: 10.1164/rccm.202009-3533SO 33146552 PMC7781116

[34] He S . Clinical observation of Chinese medicine acupoint application in the treatment of cancer induced insomnia in patients with lung cancer chemotherapy. Chin J Traditional Med Sci Technol (2021) 28(02):281–3.

[35] Hoxtermann MD Buner K Haller H Kohl W Dobos G Reinisch M . Efficacy and safety of auricular acupuncture for the treatment of insomnia in breast cancer survivors: A randomized controlled trial. Cancers (Basel) (2021) 13(16):2–16. doi: 10.3390/cancers13164082 PMC8394534 34439234

[36] Balk J Day R Rosenzweig M Beriwal S . Pilot, randomized, modified, double-blind, placebo-controlled trial of acupuncture for cancer-related fatigue. J Soc Integr Oncol (2009) 7(1):1–11. 19476729

[37] Khanghah AG Rizi MS Nabi BN Adib M Leili EKN . Effects of acupressure on fatigue in patients with cancer who underwent chemotherapy. J acupuncture meridian Stud (2019) 12(4):103–10. doi: 10.1016/j.jams.2019.07.003 31351998

[38] Lin L Zhang Y Qian HY Xu JL Xie CY Dong B . Auricular acupressure for cancer-related fatigue during lung cancer chemotherapy: a randomised trial. BMJ Support Palliat Care (2021) 11(1):32–9. doi: 10.1136/bmjspcare-2019-001937 31836594

[39] Tao L Lu D Liu T Chen F Du J . Clinical study of needling fatigue group points in treating cancer related fatigue of qi and blood deficiency. J Clin Acupuncture Moxibustion. (2020) 36(08):4–8.

[40] Guo SL LY Peng Y . Effect of acupuncture therapy on cancer related fatigue of patients with gynecological malignant tumor after chemotherapy. J Clin Acupuncture Moxibustion (2014) 30(06):67–70.

[41] Ma T Wang P Zhu J Wang S Chu J Song C . Effect of xingjian decoction combined with acupoint application on cancer fatigue of advanced gastric cancer the effect on the immune function of patients. Shaanxi J Traditional Chin Med (2020) 10(41):1410–3. doi: 10.3969/j.issn.1000-7369.2020.10.019

[42] Mao JJ Farrar JT Bruner D Zee J Bowman M Seluzicki C . Electroacupuncture for fatigue, sleep, and psychological distress in breast cancer patients with aromatase inhibitor-related arthralgia: A randomized trial. Cancer (2014) 120(23):3744–51. doi: 10.1002/cncr.28917 PMC4239308 25077452

[43] Molassiotis A Sylt P Diggins H . The management of cancer-related fatigue after chemotherapy with acupuncture and acupressure: a randomised controlled trial. Complement Ther Med (2007) 15(4):228–37. doi: 10.1016/j.ctim.2006.09.009 18054724

[44] Zhu M Chu Z Huang J Wen T Huang J Ma J . Evaluation of the clinical efficacy of topical application of shiquan DabuJiawei formula at the navel and acupressure with magnetic ear beads for cancer-related fatigue. Modern Chin Clin Med (2021) 28(01):1–6.

[45] Yu M Li D Yang G Xu Y Wang X . Effects of acupuncture on cancer-related fatigue in breast cancer patients at the rehabilitation stage: A randomized controlled trial. China Med Herald (2017) 14(19):89–93.

[46] Hu M Zhao J Gu D Xu Y Yan X . Effect of percutaneous acupoint electrical stimulation combined with emotional Release therapy on patients with lung cancer chemotherapy. J Qilu Nurs (2019) 25(19):31–3. doi: 10.3969/j.issn.1006-7256.2019.19.009

[47] Ozdemir U Tasci S . Acupressure for cancer-related fatigue in elderly cancer patients: A randomized controlled study. Altern Ther Health Med (2021). 33891570

[48] Smith C Carmady B Thornton C Perz J Ussher JM . The effect of acupuncture on post-cancer fatigue and well-being for women recovering from breast cancer: A pilot randomised controlled trial. Acupunct Med (2013) 31(1):9–15. doi: 10.1136/acupmed-2012-010228 23196311

[49] Wang XY . Effects of acupoint application combined with acupuncture and moxibustion on swallowing function and cancer-related fatigue in patients with advanced esophageal cancer. Chin J Mod Nurs (2021) 27(13):1784–8.

[50] Su YZ Xia LM . Clinical study on acupuncture for cancer-related fatigue due to spleen-kidney deficiency. Shanghai J Acu-mox (2016) 35(07):830–2. doi: 10.13460/j.issn.1005-0957.2016.07.0830

[51] Luan Y Zhang Y Pan J Yang X . Clinical observation on improving cancer-induced fatigue of patients with liver qi stagnation type breast cancer by applying shu mediation yu anshen prescription at acupoint. Yunnan J Traditional Chin Med Materia Med (2021) 42(09):62–5. doi: 10.16254/j.cnki.53-1120/r.2021.09.020. Yang YLYZJPX.

[52] Yoon HG Park H . The effect of auricular acupressure on sleep in breast cancer patients undergoing chemotherapy: A single-blind, randomized controlled trial. Appl Nurs Res (2019) 48:45–51. doi: 10.1016/j.apnr.2019.05.009 31266607

[53] Page MJ McKenzie JE Bossuyt PM Boutron I Hoffmann TC Mulrow CD . The PRISMA 2020 statement: An updated guideline for reporting systematic reviews. BMJ (2021), 1–8. doi: 10.1136/bmj.n71 PMC8008539 33781348

[54] Sonbol MB Riaz IB Naqvi SAA Almquist DR Mina S Almasri J . Systemic therapy and sequencing options in advanced hepatocellular carcinoma: A systematic review and network meta-analysis. JAMA Oncol (2020) 6(12):e204930. doi: 10.1001/jamaoncol.2020.4930 33090186 PMC7582230

[55] Higgins JP Altman DG Gotzsche PC Juni P Moher D Oxman AD . The cochrane collaboration’s tool for assessing risk of bias in randomised trials. BMJ (2011) 343:d5928. doi: 10.1136/bmj.d5928 22008217 PMC3196245

[56] Jiang XJ Yang XY Xue X Xu C Yang X Liu K . Research progress on assessment tools and evaluation indexes of cancer-induced fatigue. Chin J Nurs (2012) 47(09):859–61.

[57] Reeve BB Stover AM Alfano CM Smith AW Ballard-Barbash R Bernstein L . The piper fatigue scale-12 (PFS-12): psychometric findings and item reduction in a cohort of breast cancer survivors. Breast Cancer Res Treat (2012) 136(1):9–20. doi: 10.1007/s10549-012-2212-4 22933027 PMC3739964

[58] Xue X Xu C Yang X Liu K Zhang J Chu L . An analysis on KPS score for chemotherapy patients with cancer in liaoning province. China Cancer (2013) 22(08):635–7. doi: 10.11735/j.issn.1004-0242.2013.08.A0

[59] Huang Y Zhu M . Increased global PSQI score is associated with depressive symptoms in an adult population from the united states. Nat Sci Sleep (2020) 12:487–95. doi: 10.2147/NSS.S256625 PMC7381800 32765145

[60] Li HT Zhou YB Liu JM . The impact of cesarean section on offspring overweight and obesity: A systematic review and meta-analysis. Int J Obes (Lond). (2013) 37(7):893–9. doi: 10.1038/ijo.2012.195 23207407

[61] The Cochrane Collaboration . The cochrane collaboration, cochrane handbook for systematic reviews of interventions. London, UK: The UK Cochrane Centre, United Kingdom (2011).

[62] Liu M Zhang L Sun L . Pearson Foundations of clinical research. USA: Prentice Hall (2000).

[63] Salanti G Ades AE Ioannidis JP . Graphical methods and numerical summaries for presenting results from multiple-treatment meta-analysis: an overview and tutorial. J Clin Epidemiol (2011) 64(2):163–71. doi: 10.1016/j.jclinepi.2010.03.016 20688472

[64] Puhan MA Schunemann HJ Murad MH Li T Brignardello-Petersen R Singh JA . A GRADE working group approach for rating the quality of treatment effect estimates from network meta-analysis. BMJ (2014) 349:g5630. doi: 10.1136/bmj.g5630 25252733

[65] Hua Z Zhai F Tian J Gao C Xu P Zhang F . Effectiveness and safety of oral Chinese patent medicines as adjuvant treatment for unstable angina pectoris on the national essential drugs list of China: A protocol for a systematic review and network meta-analysis. BMJ Open (2019) 9(9):e026136. doi: 10.1136/bmjopen-2018-026136 PMC6756357 31542734

[66] Garland SN Xie SX DuHamel K Bao T Li Q Barg FK . Acupuncture versus cognitive behavioral therapy for insomnia in cancer survivors: A randomized clinical trial. J Natl Cancer Inst (2019) 111(12):1323–31. doi: 10.1093/jnci/djz050 PMC6910189 31081899

[67] Egger M Davey Smith G Schneider M Minder C . Bias in meta-analysis detected by a simple, graphical test. BMJ (1997) 315(7109):629–34. doi: 10.1136/bmj.315.7109.629 PMC2127453 9310563

[68] Salanti G Ades AE Ioannidis JPA . Using network meta-analysis to evaluate the existence of small-study effects in a network of interventions. Res Synth Methods (2012) 3(2):161–76. doi: 10.1002/jrsm.57 26062088

[69] Bower JE Ganz PA Desmond KA Bernaards C Rowland JH Meyerowitz BE . Fatigue in long-term breast carcinoma survivors: A longitudinal investigation. Cancer (2006) 106(4):751–8. doi: 10.1002/cncr.21671 16400678

[70] Minton O Stone P . How common is fatigue in disease-free breast cancer survivors? A systematic review of the literature. Breast Cancer Res Treat (2008) 112(1):5–13. doi: 10.1007/s10549-007-9831-1 18064565

[71] Haidinger R Bauerfeind I . Long-term side effects of adjuvant therapy in primary breast cancer patients: Results of a web-based survey. Breast Care (Basel) (2019) 14(2):111–6. doi: 10.1159/000497233 PMC6886114 31798383

[72] Savard J Ivers H Villa J Caplette-Gingras A Morin CM . Natural course of insomnia comorbid with cancer: an 18-month longitudinal study. J Clin Oncol (2011) 29(26):3580–6. doi: 10.1200/JCO.2010.33.2247 21825267

[73] Schmidt ME Wiskemann J Steindorf K . Quality of life, problems, and needs of disease-free breast cancer survivors 5 years after diagnosis. Qual Life Res (2018) 27(8):2077–86. doi: 10.1007/s11136-018-1866-8 29740782

[74] Hilfiker R Meichtry A Eicher M Nilsson Balfe L Knols RH Verra ML . Exercise and other non-pharmaceutical interventions for cancer-related fatigue in patients during or after cancer treatment: A systematic review incorporating an indirect-comparisons meta-analysis. Br J Sports Med (2018) 52(10):651–8. doi: 10.1136/bjsports-2016-096422 PMC5931245 28501804

[75] Lau CHY Wu X Chung VCH Liu X Hui EP Cramer H . Acupuncture and related therapies for symptom management in palliative cancer care: Systematic review and meta-analysis. Med (Baltimore) (2016) 95(9):e2901. doi: 10.1097/MD.0000000000002901 PMC4782866 26945382

[76] MacPherson H Thomas K Walters S Fitter M . The York acupuncture safety study: prospective survey of 34 000 treatments by traditional acupuncturists. BMJ (2001) 323(7311):486–7. doi: 10.1136/bmj.323.7311.486 PMC48134 11532841

[77] XN . Chinese Acupuncture and Moxibustion (Third Edition 2010) by Xinnong Cheng. Beijing: Foreign Language Press (2009).

[78] Su CX Wang LQ Grant SJ Liu JP . Chinese Herbal medicine for cancer-related fatigue: a systematic review of randomized clinical trials. Complement Ther Med (2014) 22(3):567–79. doi: 10.1016/j.ctim.2014.04.007 24906595

[79] Jia YYW Xiong Y . The interventiaon effect of acupuncture on cancer-related fatigeu and mechanism of intervention study. Asia-Pacific Traditional Med (2015) 11(07):55–7.

