# Supplementary material for: Acupuncture therapies for cancer-related fatigue: A Bayesian network meta-analysis and systematic review
Source: Front Oncol. 2023 Mar 27;13:1071326. doi: 10.3389/fonc.2023.1071326 (PMC10083363; doi:10.3389/fonc.2023.1071326)
Supplement: Supplementary file 1 [file DataSheet_1.docx]

**PubMed**

#1 "Cancer Related Fatigue"[Mesh]

#2 " Cancer Related Fatigue " [Title/Abstract] OR " Cancer Fatigue " [Title/Abstract] OR " Cancer Associated Fatigue " [Title/Abstract] OR "CRF*" [Title/Abstract] AND "Acupuncture"[Mesh] OR "Acupuncture Therapy"[Mesh])

#3 #1 OR #2

#4 "Acupuncture therapy"[Mesh]

#5 "acupuncture therapy"[Title/Abstract] OR "acupuncture"[Title/Abstract] OR "meridian*"[Title/Abstract] OR "acupoint*"[Title/Abstract] OR "electronic acupuncture"[Title/Abstract] OR "electro-acupuncture"[Title/Abstract] OR "electroacupuncture"[Title/Abstract] OR "auricular needle"[Title/Abstract] OR "scalp needle"[Title/Abstract] OR "needle"[Title/Abstract] OR "body acupuncture"[Title/Abstract] OR "manual-acupuncture"[Title/Abstract] OR " transcutaneous electrical acupoint stimulation "[Title/Abstract] OR " TEAS "[Title/Abstract]

#6 #4 OR #5

#7 "clinical trials, randomized"[Mesh Terms] OR "controlled clinical trials, randomized"[Mesh Terms] OR "clinical trials as topic"[MeSH Terms] OR "random allocation"[MeSH Terms] OR "therapeutic use"[MeSH Subheading]

#8 ("clinical"[Title/Abstract] AND "trial"[Title/Abstract]) OR "clinical trial"[Publication Type] OR "random*"[Title/Abstract]

#9 #7 OR #8

#10 #3 AND #6 AND #9

**Cochrane Library**

#1 MeSH descriptor: [Cancer Related Fatigue] explode all trees

#2 fatigue

#3 cancer OR tumor OR carcinoma OR neoplasm

#4 #2 AND #3

#5 #1 OR #4

#6 MeSH descriptor: [Acupuncture] explode all trees

#7 MeSH descriptor: [Acupuncture Therapy] explode all trees

#8 MeSH descriptor: [Auriculotherapy] explode all trees

#9 MeSH descriptor: [acupressure] explode all trees

#10 MeSH descriptor: [electroacupuncture] explode all trees

#11 (acupuncture OR electroacupuncture OR acupressure OR auriculotherapy OR point application OR TEAS) OR (manual acupuncture) OR (transcutaneous electrical acupoint stimulation) OR (TEAS)

#12 #6 OR #7 OR #8 OR #9 OR #10 OR #11

#13 random OR randomization OR randomized OR randomised OR randomly

#14 #5 AND #12 AND #13 in Trials

**EMBASE**

| No. | Query |
| --- | --- |
| #25 | #11 AND #18 AND #24 |
| #24 | #19 OR #20 OR #21 OR #22 OR #23 |
| #23 | randomly' |
| #22 | randomised' |
| #21 | randomized' |
| #20 | randomization' |
| #19 | random' |
| #18 | #12 OR #17 |
| #17 | #13 OR #14 OR #15 OR #16 |
| #16 | auriculotherapy':ab,kw,ti |
| #15 | acupressure':ab,kw,ti |
| #14 | electroacupuncture':ab,kw,ti |
| #13 | acupuncture':ab,kw,ti |
| #12 | 'acupuncture'/exp |
| #11 | #1 OR #10 |
| #10 | #4 AND #9 |
| #9 | #5 OR #6 OR #7 OR #8 |
| #8 | Cancer ':ab,kw,ti |
| #7 | neoplasm':ab,kw,ti |
| #6 | cancer':ab,kw,ti |
| #5 | tumor':ab,kw,ti |
| #4 | #2 OR #3 |
| #3 | mammary':ab,kw,ti |
| #2 | Fatigue ':ab,kw,ti |
| #1 | Cancer fatigue'/exp |
|  |  |

**Web of science**

TS=(‘Cancer Related Fatigue’ OR ‘Cancer associated Fatigue’ OR ‘Cancer Fatigue’ OR ‘CRF*’) AND TS=(‘acupuncture therapy’ OR ‘acupuncture’ OR ‘meridian*’ OR ‘acupoint*’ OR ‘point application ’ OR ‘warm acupuncture’ OR ‘transcutaneous electrical acupoint stimulation’ OR ‘moxibustion’ OR ‘electronic acupuncture’ OR ‘electro-acupuncture’ OR ‘electroacupuncture’ OR ‘auricular needle’ OR ‘needle’ OR ‘body acupuncture’ OR ‘manual-acupuncture’) AND TS=(‘random*’ OR ‘clinical’ OR ‘trial’ )
